# Vitiligo and Mental Health: Natural Compounds’ Usefulness

**DOI:** 10.3390/antiox12010176

**Published:** 2023-01-11

**Authors:** Luca Di Bartolomeo, Paolo Custurone, Natasha Irrera, Francesco Borgia, Federico Vaccaro, Francesco Squadrito, Mario Vaccaro

**Affiliations:** 1 Department of Clinical and Experimental Medicine, Dermatology, University of Messina, 98125 Messina, Italy; 2 Department of Clinical and Experimental Medicine, Pharmacology, University of Messina, 98125 Messina, Italy; 3 Department of Dermatology, University of Modena and Reggio Emilia, 41121 Modena, Italy

**Keywords:** vitiligo, natural compounds, oxidative stress, antioxidants, mental health, depression, anxiety, obsessive compulsive disorder, dermatosis, supplement

## Abstract

Vitiligo is an autoimmune dermatosis frequently associated with other comorbidities, such as mental health disorders. It is unclear if vitiligo triggers mental disorders or if mental disorders trigger vitiligo, but each one affects and worsen the other, if present at the same time. Both mental health disorders and vitiligo present a multifactorial pathogenesis and often require prolonged periods of therapy, sometimes with poor results. Given the possible link of common pathogenetic factors and the need of integrated therapies, the aim of this review is to look at natural compounds as possible supplements for both conditions. The results yielded show a possible role of these supplements in ameliorating both conditions, thus helping these patients to achieve a better quality of life and reduce the need for prolonged therapies. The limitations regarding the relative lack of in vivo studies, and the increasing need to lighten the burden of these chronic diseases, suggests that it is mandatory to proceed with further trials.

## 1. Introduction

**Figure 1:**
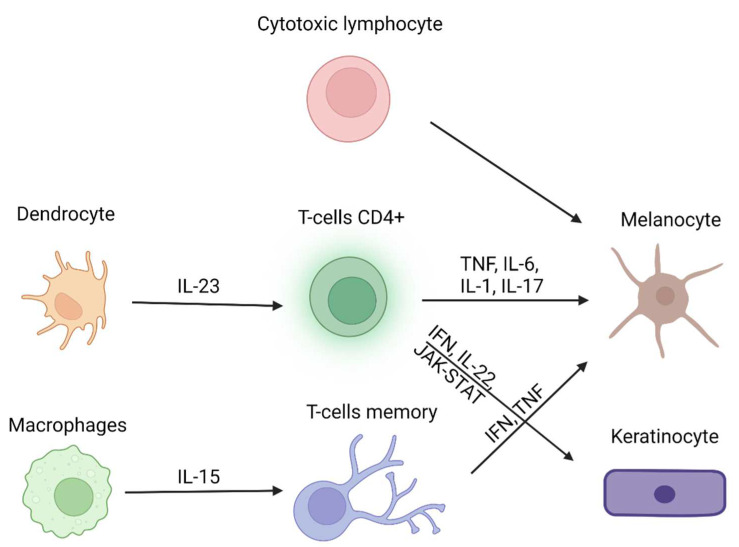
Main cell populations and cytokines involved in vitiligo (modified from Role of Cytokines in Vitiligo: Pathogenesis and Possible Targets for Old and New Treatments [3]). Created with BioRender.com (accessed on 1 November 2022).

Vitiligo is a dermatosis consisting of the appearance of white discoloration patches of the skin, with a prominent pathogenetic factor represented by autoimmunity [1]. Segmental and non-segmental are the two main forms of vitiligo, which may be recognized depending on the onset and localization [1]: the first one is the most common and affects people with autoimmune-prone habits, whereas the non-segmental vitiligo is characterized by symmetrical white macules/patches spread over the skin surface; when untreated it is usually progressive. Macules may appear close to the mucosae (mouth, orbits, genitals, anus) and flexural areas (wrists, axillae, etc.) and although they do not immediately affect physical health, they represent not only a cosmetic concern but also a risk for non-melanoma skin cancer (NMSC) development, because of the reduction of sun protection due to the lack of melanin. Different factors have been outlined as possible triggers in the development of the typical lesions, such as the tendency to develop other autoimmune diseases like Hashimoto’s thyroiditis, Addison’s disease, or alopecia areata as well as oxidative stress, trauma, sunlight exposure, and genetic factors. Several predisposing factors have been proposed in the pathogenesis of vitiligo. Individual genetic variants seem to play an important role, such as the human leukocyte antigen (HLA) cluster, the gene that encodes for the tyrosinase (TYR) or the melanocyte proliferating gene 1 (MYG1). In addition, micro-RNAs (miRNAs), strings of non-encoding RNA, have been described as possible contributors to vitiligo development, along with reduced levels of antioxidant enzymes like superoxide dismutase and glutathione peroxidase, thus supporting the hypothesis that oxidative stress is a key triggering factor. Cytokine imbalance and the activation of self-targeting immune cells have also been suggested as some of the leading causes of vitiligo. Among these, INFγ, IL-1β, CD4^+^, and CD8^+^ T-cells seem to significantly contribute to the pathogenesis of the disease. Moreover, the latest scientific evidence reports that vitiligo patients may be concomitantly affected by psychiatric disorders, possibly sharing some common pathogenetic pathways with vitiligo. Several lines of treatments have been proposed to treat this complex disease, ranging from the most common topical steroids and ultraviolet therapy to some attempts in more experimental approaches [2], although just as palliatives; moreover, compliance of patients is not always achieved [3]. A new hope is being held by new Janus kinase inhibitors (JAKi): these small molecules, via the inactivation of autoimmune and inflammatory processes, lead to the resolution of inflammatory patches but also on longstanding lesions, both alone [4] and combined with narrowband UVB-therapy [5]. Although these drugs have been demonstrated as very effective in disease control, the side effects are not trivial, the most common of which are nausea and abdominal discomfort. To help these patients, the FDA recently approved the treatment with topical ruxolitinib, a new JAK used orally for the treatment of myelofibrosis [6] and hematological tumors [7], but when applied topically leads to the re-pigmentation of previously blanched patches [8]. Nonetheless, increasing evidence shows that a person’s self-image is perceived more and more as a cardinal feature in self-perception, and normal psychological functionality and the outcomes of classical and new therapies for vitiligo show some margin of improvement in certain cases. Even though the efficacy and safety of these treatments have been tested thoroughly, and given the increasing incidence of mental health disorders in the general population, in the last decades it has been demonstrated that different compounds of natural origin show some effects that may be useful to treat both skin diseases and mental disorders. Therefore, the aim of this review is to provide an overview of the possible use of natural compounds to treat patients who suffer from vitiligo and mental disorders at the same time. Figure 1 resumes the main cell populations and cytokines involved in the autoimmune pathogenetic hypothesis, the most relevant pathway for the development of vitiligo manifestations.

## 2. Common Psychiatric Disorders Associated with Vitiligo

**Figure 2:**
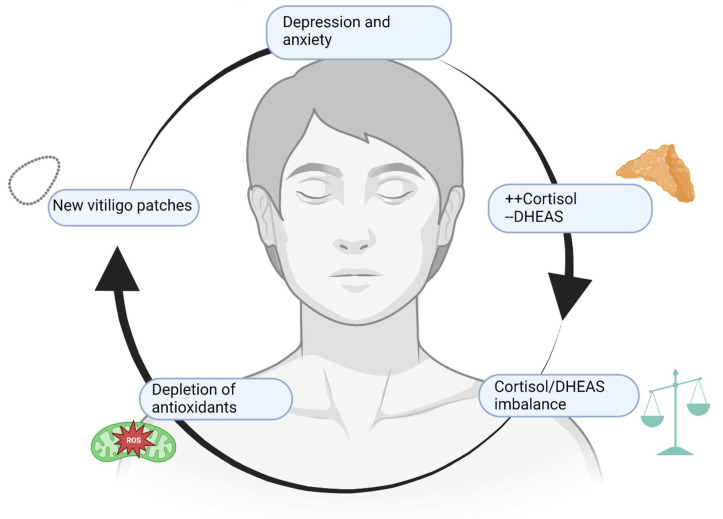
A vicious cycle. Depression and anxiety, along with other MH issues, raise cortisol levels and reduce DHEAS levels. The consequent imbalance leads to a reduction of defense mechanisms and redox potential, heightening local and systemic levels of inflammation with worsening and multiplication of vitiligo patches. If located on exposed or intimate areas, vitiligo lesions lead to stress and concern about physical appearance, worsening depressive and anxiety symptoms. Created with BioRender.com (accessed on 1 November 2022).

Patients affected by vitiligo present an increased risk of developing psychiatric disorders. Anxiety and depression represent the main mental conditions related to vitiligo, but some scientific evidence reported that other disorders such as obsessive–compulsive disorder (OCD), maniac disorder, bipolar disorder, and schizophrenia may concomitantly affect vitiligo patients [9]. OCD and schizophrenia share a common pathogenetic ground with vitiligo, probably because of autoimmune and inflammatory involvement, as an increase in pro-inflammatory cytokines such as IL-1, IL-6, and TNF-α has been found in both groups of patients [9]. The same association has been previously hypothesized with psoriasis, which is classically related to stress, anxiety, and depression. However, vitiligo seems to have a stronger correlation with mental disorders than that observed in psoriatic patients [10], as 90% of vitiligo-affected patients are also diagnosed with at least one psychiatric disorder [11]. Anxiety represents the most frequent disorder, but up to 40% of patients also showed depressive symptoms. Independently from the variety and severity of mental health (MH) conditions that afflict these patients, the cost that derives from hospitalization represents an increasing economic burden on public health: 4% of vitiligo/MH patients required hospitalization, versus 2% of non-vitiligo/MH patients, with a total of ≃$10 million excess annual costs for hospitalization in a 10-year period [12]. A specific correlation between vitiligo and MH disorders has not been found, although common genetic and molecular backgrounds may be responsible for this combined appearance. For instance, a study conducted in India showed that MH disorders were most common in vitiligo patients [10], whereas a more recent study defined that psoriasis affects the quality of life (QoL) of patients more than vitiligo [13]. The different results could be attributed to sample size or to differences in the setting of the experimental studies, therefore additional studies are required to confirm these data. In fact, another study that assessed the QoL of vitiligo patients demonstrated that even if patients showed depressive–anxious symptoms, QoL was not so much impaired; it depended on patient behavior, self-perception, age and symptoms appearance [14]. Nonetheless, the psychological consequences of vitiligo-affected patients are certain, and psychological issues and disease awareness should be addressed by dermatologists [15]. Vitiligo patients can develop MH disorders later in life because of their condition, slowly developing when unmet needs pile up: according to a study, patients affected by vitiligo develop sexual uneasiness due to their condition, leading to scarce sexual arousal and feelings of insecurity with anxiety [16]. This feeling of insecurity and self-consciousness is measured with Beck’s Anxiety Inventory and Beck’s Depression Inventory, and patients affected by vitiligo scored higher than healthy controls [17]. Anxiety and stress levels are higher in patients affected by vitiligo and acne, especially if visible areas are involved. In fact, patients affected by vitiligo are more prone to lead a solitary life, engaging in avoidant behavior due to their pathology with consequent social anxiety, difficulties at work or school, and romantic relationship struggles. In time, if no other coping strategy is taken, depressive symptoms may occur until a depressive syndrome can manifest [18]. Stressful life events are more common in vitiligo-affected children, and symptoms of depression can be noticed as well, although they are not as common as in other autoimmune conditions such as alopecia areata [19]. Children, more than adolescents, are prone to develop depressive and anxiety symptoms when affected by vitiligo, and the reasons can be multiple: it has been suggested that exposed areas are less easily covered by makeup than in adults or adolescents, making these children more self-conscious about their physical aspect. On the other hand, dyschromic lesions in the genital area can change sexual perception and development in child patients more than in adults, suggesting that both visible and invisible areas can damage self-perception [20]. In adolescents, the duration of the disease and the affected areas seem to be the most reliable factors for psychiatric symptoms development, especially considering that this is the age of first sexual experiences. Although there is no specific cut-off that can suggest how much of the body’s surface or which areas are the most affected in adolescents, extension and visible areas are directly proportional to psychological impairment. Future studies are needed to extract more precise data on the correlation between MH issues and vitiligo severity, perhaps via standardized questionnaires [21]. A possible molecular explanation of common pathogenesis of MH disorders and vitiligo can be also found in stress hormones. Although stress hormones do not represent the first pathogenetic markers for vitiligo manifestations, levels of cortisol and dehydroepiandrosterone (DHEAS) and their ratio (+cortisol/−DHEAS) are related to vitiligo severity and increase the prevalence of psychiatric issues. DHEAS is considered an antioxidant hormone that could play a role in the appearance of vitiligo lesions and its levels increase during oxidative stress, thus making this hormone a possible future marker of vitiligo activity or severity, and a predictive factor for anxiety and depression [22]. Therefore, vitiligo may have some connection with MH disorders of the depressive spectrum, and the prognosis of the diseases could depend on each other if present in the same patient. Figure 2 resumes how MH disorders and vitiligo interlace via the effects of common mediators and hormones.

## 3. Current Therapies for Depression and Vitiligo

As of today, several drugs have been proposed to treat both depressive syndromes and vitiligo, with great overall efficacy. However, this still burdens patients with the need for constant prescriptions and an overall increase of side effects related to their use. Antidepressants include monoamine oxidase inhibitors (MAOIs) and tricyclic antidepressants (TCAs) such as amitriptyline. These drugs were then followed by selective serotonin reuptake inhibitors (SSRIs) such as fluoxetine, serotonin/norepinephrine reuptake inhibitors (SNRIs) such as venlafaxine, norepinephrine/dopamine reuptake inhibitors (NDRIs) such as bupropion, serotonin antagonist-reuptake inhibitors (SARIs) such as trazadone, agents with indirect noradrenergic and serotonergic actions (NaSSAs) such as mirtazapine, and agents with multimodal serotonergic targets (vilazodone and vortioxetine) [23]. Their effects are mostly attributed to the enhancement of monoaminergic functions and selective serotonin and norepinephrine reuptake inhibition, thus increasing neurotransmitter concentration in the synaptic space. Recently, a revival of psychedelic drugs has been taking place, with drugs like psilocybin [24] or ayahuasca [25] which could activate the serotonergic receptors, thus modifying amygdala reactivity and enhancing hippocampal neuroplasticity. However, the use of these approaches is related to various side effects and can lead to treatment discontinuation. MAOIs and TCAs can be lethal due to cardiac toxicity, and require constant monitoring of the Q-T interval by psychiatrists [26]. Nausea, sexual dysfunction (loss of desire or anorgasmia), insomnia, somnolence, fatigue, bruxism, and weight gain are commonly found in the case of SSRIs and SNRIs therapy, while a sudden stop to the therapy can lead to withdrawal symptoms like anxiety, irritability, and nausea [27]. All of these and other adverse events are pushing researchers to find more tolerable substances, such as natural compounds, which might help patients wean from chronic therapies and thus reaching better compliance. As with depression, different therapeutic approaches are used for the treatment of vitiligo, aiming at re-pigmentation, or reducing inflammation that causes pigment loss; usually, however, these are chronic and long-lasting therapies. In this context, the prolonged application of medium to high potency corticosteroids leads to hyperpigmentation and significantly reduces inflammation, but can lead to skin atrophy. Calcineurin inhibitors also represent a good option for the treatment of visible areas, but they are not always efficient and have a scarce therapeutic adherence. In fact, new treatments are under consideration, such as vitamin D analogs, which showed efficacy when used in association with other treatments; superoxide dismutase/pseudocatalase creams showed comparable results to calcineurin inhibitors, but no safety profile has been tested thoroughly yet; 5-fluorouracil, which demonstrated a good re-pigmentation rate but can lead to scarring and infections if not properly handled; JAK inhibitors, in particular ruxolitinib and tofacitinib, have a good safety profile with a lower percentage of efficacy in monotherapy than that observed when combined with narrow band UVB-therapy (nb-UVB). Systemic therapies are mainly used in patients with early or progressive disease. Nb-UVB is administered two to three times weekly, and it is usually used for up to one year. Oral glucocorticoids, mainly daily prednisone, are used to stabilize the progressive disease with a short course lasting two to six weeks. Alternatively, pulse therapy may be useful for two days/week for three months. Finally, intramuscular triamcinolone is used with a single administration weekly for a maximum of three weeks. Glucocorticoids should be associated with phototherapy to achieve re-pigmentation. Other immunosuppressants have been attempted with variable results, and recently biological drugs targeting cytokines involved in vitiligo pathogenesis are under investigation [3].

## 4. Natural Compounds Used for the Treatment of Patients Affected by Vitiligo and Mental Disorders

The anti-inflammatory and mood-regulating properties of natural compounds have been recently described for psoriasis patients [28]. Natural compounds with antioxidant, anti-inflammatory, and neuroprotective properties may also be useful for the management of vitiligo patients, in whom oxidative stress, autoimmunity, and mental disorders are associated in a complex relationship. In the next section, the main pathogenetic pathways of vitiligo and mental disorders targeted by natural compounds will be described.

### 4.1. Main Pathogenetic Pathways of Vitiligo and Mental Disorders Targeted by Natural Compounds 

Oxidative stress, autoimmunity, and alteration of melanogenesis are the main pathogenetic mechanisms of vitiligo, and natural compounds counter one or more of these. The overproduction of reactive oxygen species (ROS) and the deficiency of antioxidants enzymes cause an imbalance of cellular redox status and consequently damage melanocytes [29]. Different molecules and pathways targeted by natural compounds are involved in oxidative stress, such as the nuclear factor erythroid 2-like factor 2 (Nrf2), which improves the antioxidant activity of melanocytes. The regulation of melanogenesis and tyrosinase activity is essential in the treatment of vitiligo; melanin production, as well as its transport, may be stimulated by several cytokines, including the α-melanocyte-stimulating hormone (α-MSH) and stem cell factor (SCF). These factors express their signal through several molecular pathways, such as phosphatidylinositol-3-kinase and protein kinase B (PI3K/AKT) or p38 MAP kinase, which converge towards an increase of microphthalmia-associated transcription factor (MITF) expression, thus enhancing RNA levels of tyrosinase (TYR), tyrosine-related protein-1 (TRP-1) and tyrosine-related protein-2 (TRP-2), which produce melanogenesis in melanosomes [30] that can be regulated by natural compounds. One of the most important cytokines involved in vitiligo is INFγ which promotes the skin homing of melanocyte-specific CD8^+^ cytotoxic T lymphocytes (CTLs) and induces the production of several chemokines, particularly CXCL10. INFγ effects are mediated by the Janus kinase and Signal Transducer and Activator of Transcription (JAK/STAT) pathway [3]. In addition to INFγ, other cytokines contributing to inflammation in vitiligo, such as TNF-α or IL-1β, may be targeted by natural compounds that may have not only well-known anti-inflammatory effects, but may also manage mental disorders. In fact, neuroinflammation represents one of the main causes of mental disorders such as major depression, anxiety, and schizophrenia [31,32]. Innate inflammation and Th1-Th2 cytokine imbalance may activate glial cells, neurotrophism and may affect neurotransmitter levels [33]. Neuroinflammation is characterized by an increase of pro-inflammatory cytokines, such as IL-1β, IL-2, IL-6, TNF-α, and IFN-γ which are produced by microglia, Th1 lymphocytes, and M1 phenotype monocytes/macrophages. One of the pathogenetic theories of depression relates to the monoamine hypothesis, according to which depressive symptoms are caused by the depletion of serotonin, norepinephrine, and/or dopamine levels in the central nervous system [34]. Low levels of the neurotransmitter GABA, as well as the increase of glutamate linked to depression and the use of some natural compounds, may also be useful in regulating these neurotransmitters. In addition, the alteration of the neurotrophic activity of the brain-derived neurotrophic factor (BDNF) may contribute to depression [35]. BDNF stimulates neurogenesis, synaptic plasticity, and neurotransmission. In fact, the reduction of BDNF levels is considered to be an important cause of depressive symptoms [36], therefore BDNF may also be considered an important target for the treatment of depression, and some natural compounds may induce its expression. Additionally, the endocannabinoid system may be targeted by natural compounds, thus increasing serotonin and GABA levels. In the following sections, the description of natural compounds’ efficacy will be reported for the treatment of vitiligo and mental disorders.

### 4.2. Baicalein

Baicalein is a flavonoid deriving from *Scutellaria baicalensis Georgi* whose anti-inflammatory and antioxidant effects have been demonstrated [36]. In vitiligo-affected melanocytes, the activation of antioxidant mechanisms, such as that of the nuclear factor erythroid 2-like factor 2 (Nrf2), is impaired [36]. Ndf2 is a transcription factor that activates antioxidant and detoxification genes such as heme oxygenase-1 (HO-1) or superoxide dismutase (SOD) in response to oxidative stress [36], which plays an important role in the pathogenesis of vitiligo [37]. As demonstrated by in vitro experiments, Baicalein may upregulate the Nrf2 signaling pathway in human melanocytes, thus protecting cells from oxidative stress [36]. Moreover, other experimental models showed that Baicalein may exert anti-depressant effects by regulating neurogenesis [38]; in particular, Baicalein may upregulate extracellular signal-regulated kinase (pERK) phosphorylation and BDNF. As with BDNF, ERK, a member of the mitogen-activated protein kinases (MAPKs) family, also has neurotrophic properties and is involved in the neurogenesis of the hippocampus [38]. The activation of these factors would have an anti-depressant effect; also, fluoxetine, an antidepressant drug, acts through ERK1/2 phosphorylation (p-ERK1/2) regulation in the hippocampus.

### 4.3. Quercetin

Quercetin is a polyphenolic flavonoid found in fruits such as apples and cranberries, vegetables such as onion and asparagus, and herbs including dill, cilantro, and Camellia sinensis (black tea). Quercetin has anti-inflammatory, antioxidant, anti-cancer as well as neuroprotective properties [39] and seems to be also protective in vitiligo patients. Previous results indicated that quercetin may prevent endoplasmic reticulum (ER) swelling induced by oxidative stress [40], and may modulate the inhibition of tyrosinase observed in human epidermal melanocytes [40]. In fact, like other flavonoids, quercetin stimulates melanogenesis by increasing intracellular tyrosinase activity [41] and melanin in melanoma cells in a dose-dependent manner [42]. Flavonoids influence melanogenesis by activating the MITF, which regulates the expression of the most important melanogenic enzymes, such as tyrosinase, dopachrome tautomerase (DTC, also known as tyrosine-related protein 2, TYRP-2), and tyrosine-related protein 1 (TYRP-1) [43]. Quercetin may be useful for the treatment of psychiatric disorders, as demonstrated by Samad et al. who evaluated the effect of a parenteral administration in anxious and depressed mice: quercetin regulated serotonergic and cholinergic neurotransmission of mice, contrasting anxiety and depression, but also improved memory performance [44]. Moreover, thanks to its antioxidant activity, quercetin can modulate the altered expression of phosphoinositide 3-kinase (PI3K), protein kinase B (Akt), Nrf2 and heme oxygenase-1 (HO-1) observed in depression [45].

### 4.4. Kaempferol

Kaempferol is a flavonoid found in many vegetables and fruits as well as plants and herbs, which is well known for its anti-tumor properties [46] thanks to its ability in inhibiting malignant proliferations [47,48]. Previous studies indicated that Kaempferol may promote melanogenesis, although the involved mechanisms are not well-known [49]. Kaempferol was able to promote melanogenesis, melanosome maturation, and melanin transport from perinuclear to dendritic tips of melanocytes [49], and as quercetin, kaempferol may stimulate melanogenesis in a dose-dependent manner [50]. These effects would be mediated by p38/ERK/MAPK phosphorylation and PI3K/AKT signaling downregulation [49]. Together with its anti-cancer effects, kaempferol has neuroprotective properties. For this reason, it has been tested and studied in different neurodegenerative diseases such as Parkinson’s and Alzheimer’s disease, as well as for the management of depression and anxiety [45]. The antidepressant effects of kaempferol were related to its antioxidant and anti-inflammatory effects through the modulation of different pathways such as AKT and β-catenin and reducing TNF-α and IL-1β levels [50]. The anxiolytic action of kaempferol is supported by in vitro and in vivo studies [51] that demonstrated an anti-anxiety activity like diazepam. The anxiolytic effect of kaempferol is also related to endocannabinoids levels regulation as anandamide, which plays significant anxiolytic effects: kaempferol inhibits the fatty-acid amide hydrolase (FAAH), an enzyme that catabolizes anandamide, in a concentration-dependent manner, thus elevating the levels of anandamide [51].

### 4.5. Epigallocatechin-3-Gallate

Epigallocatechin-3-Gallate (EGCG) is a polyphenolic catechin mostly contained in green tea (*Camellia sinensis*). EGCG has anti-cancer, antioxidant, anti-inflammatory, and anti-infective effects, although its low bioactivity after oral administration restricts its use [52,53]. A topical ointment with EGCG is already licensed for the treatment of external genital warts [52,54], whereas its topical use for vitiligo treatment is still being investigated [55]. In a recent randomized controlled trial, EGCG positive effects on re-pigmentation were observed in vitiligo patients using pimecrolimus ointment [55]. Nevertheless, EGCG does not seem to have a direct melanogenesis-promoting action, even though it might reduce melanin synthesis by inhibiting tyrosinase accumulation [40,42]. Therefore, the usefulness of EGCG in vitiligo patients would not be related to its effects on melanogenesis, but to its antioxidant and anti-inflammatory properties [40,56]. Peracetylated EGCG is the derivative with a greater bioavailability, whose antioxidant effect was observed in human epidermal melanocytes by reducing ROS production [57]. Moreover, the topical administration of EGCG in mice with monobenzone-induced vitiligo reduced serum levels of the pro-inflammatory cytokines TNF-α, IFN-γ, and IL-6, as well as the perilesional CD8+ T cells accumulation [56]. The reduced expression of IFNγ decreases, in turn, the downstream targets JAK2 and STAT1/3, which are particularly involved in the pathogenesis of vitiligo [58]. Moreover, EGCG may reduce IFNγ-induced chemokines such as CXCL10, and the expression of the related receptors including CD11a, CXCR3, and CCR2 in human T lymphocytes [58]. In relation to this evidence, experimental in vivo studies have highlighted the promising properties of EGCG in neuromodulation, thus ameliorating depression-related behaviors and enhancing serotonin levels in the hippocampus [59,60,61]. Moreover, EGCG can decrease IL-6 levels and its downstream transcription factor STAT3 in the hippocampus, counteracting neuroinflammation and reducing anxiety-like behaviors [60]. The anxiolytic properties of EGCG are also related to the modulation of gamma-aminobutyric acid (GABA) receptors and to the inhibition of spontaneous excitatory synaptic transmission [61]. In contrast to its usefulness in depression and anxiety, EGCG did not show antipsychotic effects in patients with schizophrenia and bipolar disorder [62]: no significant difference was observed between placebo and EGCG -treated groups in a double-blind, randomized controlled trial on patients with schizophrenia, schizoaffective disorder, or bipolar disorder [62].

### 4.6. Curcumin

Curcumin is a polyphenolic compound taken from turmeric (*Curcuma longa*) which, like the other natural compounds described so far, shows anti-inflammatory, antimicrobial, antioxidant, and anti-neoplastic properties [63]. These pleiotropic effects have raised interest also in the field of dermatology and phytotherapy, so an application for vitiligo was hypothesized. A combination treatment composed of a tetrahydrocurcuminoid cream plus narrowband UVB phototherapy was used in patients suffering from focal or generalized vitiligo. The combination treatment did not improve re-pigmentation compared to the group that received phototherapy only, although this result may be explained by the small sample size [64]. To the best of our knowledge, no studies reported the possible efficacy of systemic administration of curcumin on vitiligo in animals or humans. Curcumin may activate the Nrf-2 signaling pathway, which is impaired in vitiligo, upregulating antioxidant and detoxification genes and protecting cells from oxidative stress [65]. Moreover, curcumin may inhibit many pro-inflammatory molecules, including IFNγ, which play a critical role in the pathogenesis of vitiligo [66]. The ability of curcumin in inhibiting IFNγ was already demonstrated in psoriasis but was not demonstrated in vitiligo [66], even if curcumin was able to inhibit melanogenesis in an in vitro model, thus reducing melanin content and tyrosinase activity in a dose-dependent manner [67]. The poor bioavailability of curcumin represents the main limitation of its possible use. Therefore, different experimental approaches are aimed at testing new formulations to ameliorate its bioavailability: modified curcumin suppressed melanogenesis by activating the extracellular signal-regulated protein kinase (ERK) pathway [68]. Curcumin supplementation is an efficient alternative treatment for depressive and anxiety symptoms, thus improving the quality of life of patients with chronic disorders [69]. However, the available evidence of curcumin’s effects on vitiligo is still contrasting, therefore new data are required to provide a recommendation for its use in the clinical practice for vitiligo management.

### 4.7. Cannabidiol

Cannabidiol (CBD) is a non-psychoactive compound derived from the *Cannabis sativa* L. which, compared to ∆9-*trans*-tetrahydrocannabinol (the main compound extracted from the plant), does not induce intoxication and is not considered a psychoactive drug [70]. Increasing interest has been raised about CBD neuroprotective effects which are currently used for the treatment of refractory epilepsy in children [71]. Moreover, both animal and human studies have shown promising results concerning CBD use for the treatment of depression, anxiety, and psychotic disorders, such as schizophrenia [70]. CBD was also used in many skin disorders, although evidence concerning its use in vitiligo is still lacking [72]. However, the data obtained so far in other experimental models indicated that CBD may protect against oxidative stress by preventing free radical formation and activating Nrf2, improving antioxidant enzyme transcription [73]. Moreover, CBD has significant anti-inflammatory effects, thus reducing pro-inflammatory cytokines release and inhibiting T cell proliferation [73]. Nevertheless, the relationship between melanogenesis and the role of cannabinoids is not completely clear: cannabinoid-1 (CB-1) receptor agonism may induce different responses in melanogenesis, inducing both reduction and induction of this process [74,75]. CBD may play a role as adjuvant therapy in vitiligo thanks to its antioxidant and anti-inflammatory effects, although its effect should be fully elucidated in melanogenesis. Moreover, cannabidiol-related adverse events should not be underestimated: they may include somnolence, gastrointestinal disorders, an increase in liver function, and drug interactions [76].

### 4.8. Glycyrrhizin and Glycyrrhetinic Acid

**Figure 3:**
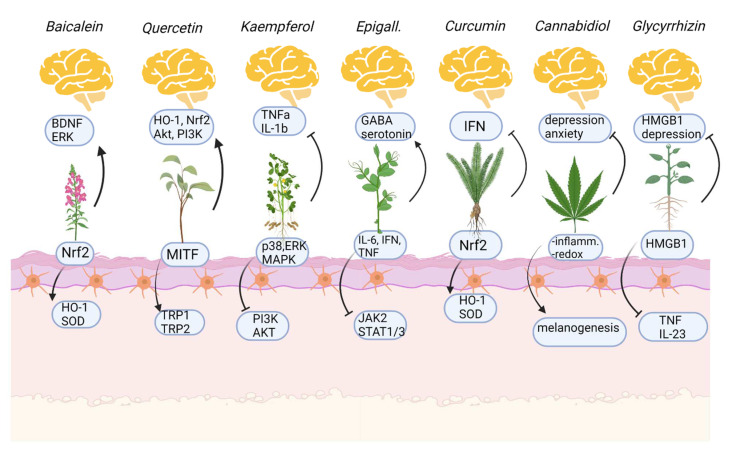
Mechanism of action of natural compounds on the skin (below, melanocytes) and on mental health issues (above). Arrows indicate activation, stops indicate inhibition. Created with BioRender.com (accessed on 1 November 2022).

Glycyrrhizin is a triterpenoid saponin glycoside extracted from licorice (*Glycyrrhiza glabra*), composed of one glycyrrhetinic acid (GA) and two glucuronic acids [77]. Glycyrrhizin has anti-inflammatory, antioxidant, and antiviral activity; in fact, has been recently proposed as an adjunctive treatment for the SARS-CoV-2 infection [78]. The anti-inflammatory effects of glycyrrhizin are related to its ability in inhibiting the high-mobility group box-1 gene (HMGB1), which stimulates pro-inflammatory cytokines production, including TNFα [79] and IL-23 [80]. Moreover, glycyrrhizin was found to protect melanocytes from oxidative stress by inducing the nuclear translocation of Nrf2 in human melanocytes, thus inducing the expression of HO-1, an antioxidant enzyme responsible for heme degradation [81]. The effects of glycyrrhizin on melanocytes also involve the stimulation of melanogenesis: glycyrrhizin may increase tyrosinase mRNA levels as well as TRP-2 expression and melanin content in a dose-dependent manner [77]. In addition, Lee et al. demonstrated that glycyrrhizin may stimulate melanogenesis with a mechanism of action that involves cAMP signaling activation [82]. The oral administration of glycyrrhizin in association with UVB irradiation caused re-pigmentation of lesions in 87.5% of patients, with no appearance of new lesions in previously active vitiligo [83]. Glycyrrhizin also showed anti-depressant effects in patients which were related to its anti-inflammatory properties. The symptomatic improvement was higher in patients that showed high levels of inflammatory markers at baseline [84], even if animal models of depression have shown that the antidepressant activity of glycyrrhizin lies in its ability to block inflammation induced by HMGB1, which is responsible for depressive behaviors in mice [85,86] and the production of IL-33 [87], which also has been demonstrated as an interleukin significantly overexpressed in vitiligo-affected patients [88]. Moreover, glycyrrhizin may regulate neurotransmitter levels in the amygdala of mice which showed a significant alteration of the circadian rhythm of serotonin [89]. Glycyrrhizin may normalize the serotonin fluctuations, thus demonstrating an interesting potential for the treatment of anxiety and stress-related disorders [89]. Effects of natural compounds on the skin and the brain are resumed in Figure 3.

## 5. Conclusions

Vitiligo is a complex disease whose pathogenesis results from the interaction of different factors, including genetic predisposition as well as oxidative and psychological stress. Vitiligo patients show a high risk of developing psychiatric disorders, in particular anxiety and depression, which may be the psychological consequences of social embarrassment and/or aggravating factors of the skin disease. Natural compounds may be useful and safe treatment options in patients with inflammatory skin disease and mental disorders, considering their anti-inflammatory and mood-regulating effects which have been already demonstrated in other skin diseases, such as psoriasis. However, few RCTs have been conducted on the use of natural compounds in vitiligo patients. Most of the studies include in vitro and in vivo experimental models aimed at evaluating the effects of natural compounds on melanogenesis, autoimmunity, and mental health. These data support the future development of clinical trials to better investigate the therapeutic potential for a possible application in the clinical practice for patients with vitiligo and mental disorders. Table 1 offers a small recap of the current evidence of the benefits derived from natural compounds, both on vitiligo and on MH disorders.

## Figures and Tables

**Table 1:** Natural compounds tested both on vitiligo and mental health issues with respective effects.

Natural Compound	Effects on Vitiligo	Effects on Mental Health
***Baicalein***	- lowers levels of inflammation and oxidation	- enhances effects neurotrophic and neuroprotective factors
***Quercetin***	- increase tyrosinase and MITF activation	- modulation of antioxidant genes
***Kaempferol***	- enhances melanogenesis and melanosomes’ migration to the dendrites of melanocytes	- lowers levels of inflammation
***Epigallocatechin***	- lowers inflammation levels - reduction of pro-inflammatory cytokines - reduces CD8^+^ infiltration	- nhibition of IL-6 - modulation of GABA secretion and reduction of anxiety and depression-related symptoms
***Curcumin***	- not enough studies on vitiligo - modified curcumin potentially not ameliorating vitiligo	- ameliorates anxiety and depressive symptoms
***Cannabidiol***	- potential reduction of oxidative stress (more studies needed)	- lowers levels of stress and anxiety
***Glycyrrhizin***	- inhibitor of inflammation - enhancement of melanogenesis - more effective when combined with nb-UVB therapy	- reduction of inflammation - enhances production of serotonin

## References

[1] Ezzedine K., Eleftheriadou V., Whitton M., van Geel N. (2015). Vitiligo. Lancet.

[2] Di Bartolomeo L., Altavilla D., Vaccaro M., Vaccaro F., Squadrito V., Squadrito F., Borgia F. (2022). Photodynamic therapy in pediatric age: Current applications and future trends. Front. Pharmacol..

[3] Custurone P., Di Bartolomeo L., Irrera N., Borgia F., Altavilla D., Bitto A., Pallio G., Squadrito F., Vaccaro M. (2021). Role of Cytokines in Vitiligo: Pathogenesis and Possible Targets for Old and New Treatments. Int. J. Mol. Sci..

[4] Rosmarin D., Passeron T., Pandya A.G., Grimes P., Harris J.E., Desai S.R., Lebwohl M., Ruer-Mulard M., Seneschal J., Wolkerstorfer A. (2022). Two Phase 3, Randomized, Controlled Trials of Ruxolitinib Cream for Vitiligo. New Engl. J. Med..

[5] Yousefian F., Yadlapati S., Browning J.C. (2022). The use of Janus kinase inhibitors and narrowband ultraviolet B combination therapy in non-segmental vitiligo. J. Cosmet. Dermatol..

[6] Guglielmelli P., Kiladjian J.-J., Vannucchi A.M., Duan M., Meng H., Pan L., He G., Verstovsek S., Boyer F., Barraco F. (2022). Efficacy and safety of ruxolitinib in patients with myelofibrosis and low platelet count (50 × 109/L to <100 × 109/L) at baseline: The final analysis of EXPAND. Ther. Adv. Hematol..

[7] Van Den Neste E.V.D., André M., Gastinne T., Stamatoullas A., Haioun C., Belhabri A., Reman O., Casasnovas O., Ghesquieres H., Verhoef G. (2018). A phase II study of the oral JAK1/JAK2 inhibitor ruxolitinib in advanced relapsed/refractory Hodgkin lymphoma. Haematologica.

[8] Sheikh A., Rafique W., Owais R., Malik F., Ali E. (2022). FDA approves Ruxolitinib (Opzelura) for Vitiligo Therapy: A breakthrough in the field of dermatology. Ann. Med. Surg..

[9] Chen C.-Y., Wang W.-M., Chung C.-H., Tsao C.-H., Chien W.-C., Hung C.-T. (2020). Increased risk of psychiatric disorders in adult patients with vitiligo: A nationwide, population-based cohort study in Taiwan. J. Dermatol..

[10] Mattoo S.K., Handa S., Kaur I., Gupta N., Malhotra R. (2001). Psychiatric morbidity in vitiligo and psoriasis: A comparative study from India. J. Dermatol..

[11] Ucuz I., Altunisik N., Sener S., Turkmen D., Kavuran N.A., Marsak M., Colak C. (2020). Quality of life, emotion dysregulation, attention deficit and psychiatric comorbidity in children and adolescents with vitiligo. Clin. Exp. Dermatol..

[12] Patel K., Singam V., Rastogi S., Lee H., Silverberg N., Silverberg J. (2018). Association of vitiligo with hospitalization for mental health disorders in US adults. J. Eur. Acad. Dermatol. Venereol..

[13] Karelson M., Silm H., Kingo K. (2013). Quality of Life and Emotional State in Vitiligo in an Estonian Sample: Comparison with Psoriasis and Healthy Controls. Acta Derm. Venereol..

[14] Krüger C., Schallreuter K. (2015). Stigmatisation, Avoidance Behaviour and Difficulties in Coping are Common among Adult Patients with Vitiligo. Acta Derm. Venereol..

[15] Osman A., Elkordufani Y., Abdullah M. (2009). The psychological impact of vitiligo in adult Sudanese patients. Afr. J. Psychiatry.

[16] Sukan M., Maner F. (2007). The Problems in Sexual Functions of Vitiligo and Chronic Urticaria Patients. J. Sex Marital. Ther..

[17] Hamidizadeh N., Ranjbar S., Ghanizadeh A., Parvizi M.M., Jafari P., Handjani F. (2020). Evaluating prevalence of depression, anxiety and hopelessness in patients with Vitiligo on an Iranian population. Health Qual. Life Outcomes.

[18] Salman A., Kurt E., Topcuoglu V., Demircay Z. (2016). Social Anxiety and Quality of Life in Vitiligo and Acne Patients with Facial Involvement: A Cross-Sectional Controlled Study. Am. J. Clin. Dermatol..

[19] Erdoğan S.S., Gür T.F., Doğan B. (2020). Anxiety and depression in pediatric patients with vitiligo and alopecia areata and their parents: A cross-sectional controlled study. J. Cosmet. Dermatol..

[20] Bilgiç Ö., Bilgiç A., Akiş H.K., Eskioğlu F., Kiliç E.Z. (2010). Depression, anxiety and health-related quality of life in children and adolescents with vitiligo. Clin. Exp. Dermatol..

[21] Choi S., Kim D.-Y., Whang S.-H., Lee J.-H., Hann S.-K., Shin Y.-J. (2010). Quality of life and psychological adaptation of Korean adolescents with vitiligo. J. Eur. Acad. Dermatol. Venereol..

[22] Gürpinar A., Günaydin S.D., Kiliç C., Karaduman A. (2019). Association of serum cortisol and dehydroepiandrosterone sulfate (DHEAS) levels with psychological stress in patients with vitiligo. Turk. J. Med Sci..

[23] Gonda X., Dome P., Neill J.C., Tarazi F.I. (2021). Novel antidepressant drugs: Beyond monoamine targets. CNS Spectrums.

[24] Goldberg S.B., Pace B.T., Nicholas C.R., Raison C.L., Hutson P.R. (2020). The experimental effects of psilocybin on symptoms of anxiety and depression: A meta-analysis. Psychiatry Res..

[25] Davis A.K., So S., Lancelotta R., Barsuglia J.P., Griffiths R.R. (2019). 5-methoxy-N,N-dimethyltryptamine (5-MeO-DMT) used in a naturalistic group setting is associated with unintended improvements in depression and anxiety. Am. J. Drug Alcohol Abus..

[26] Baldessarini R.J., Brunton L.L., Lazo J.S., Parker K.L. (2005). Drug therapy of depression and anxiety disorders. Goodman and Gilman’s: The Pharmacological Basis of Therapeutics.

[27] Hu X.H., Bull S.A., Hunkeler E.M., Ming E., Lee J.Y., Fireman B., Markson L.E. (2004). Incidence and Duration of Side Effects and Those Rated as Bothersome with Selective Serotonin Reuptake Inhibitor Treatment for Depression. J. Clin. Psychiatry.

[28] Alesci A., Lauriano E.R., Fumia A., Irrera N., Mastrantonio E., Vaccaro M., Gangemi S., Santini A., Cicero N., Pergolizzi S. (2022). Relationship between Immune Cells, Depression, Stress, and Psoriasis: Could the Use of Natural Products Be Helpful?. Molecules.

[29] Vaccaro M., Bagnato G., Cristani M., Borgia F., Spatari G., Tigano V., Saja A., Guarneri F., Cannavò S.P., Gangemi S. (2017). Oxidation products are increased in patients affected by non-segmental generalized vitiligo. Arch. Dermatol. Res..

[30] Niu C., Aisa H.A. (2017). Upregulation of Melanogenesis and Tyrosinase Activity: Potential Agents for Vitiligo. Molecules.

[31] Zheng Z.-H., Tu J.-L., Li X.-H., Hua Q., Liu W.-Z., Liu Y., Pan B.-X., Hu P., Zhang W.-H. (2020). Neuroinflammation induces anxiety- and depressive-like behavior by modulating neuronal plasticity in the basolateral amygdala. Brain Behav. Immun..

[32] Na K.-S., Jung H.-Y., Kim Y.-K. (2014). The role of pro-inflammatory cytokines in the neuroinflammation and neurogenesis of schizophrenia. Prog. Neuropsychopharmacol. Biol. Psychiatry.

[33] Najjar S., Pearlman D.M., Alper K., Najjar A., Devinsky O. (2013). Neuroinflammation and psychiatric illness. J. Neuroinflamm..

[34] Delgado P.L. (2000). Depression: The case for a monoamine deficiency. J. Clin. Psychiatry.

[35] Rana T., Behl T., Sehgal A., Srivastava P., Bungau S. (2020). Unfolding the Role of BDNF as a Biomarker for Treatment of Depression. J. Mol. Neurosci..

[36] Ma J., Li S., Zhu L., Guo S., Yi X., Cui T., He Y., Chang Y., Liu B., Li C. (2018). Baicalein protects human vitiligo melanocytes from oxidative stress through activation of NF-E2-related factor2 (Nrf2) signaling pathway. Free. Radic. Biol. Med..

[37] Vaccaro M., Irrera N., Cutroneo G., Rizzo G., Vaccaro F., Anastasi G.P., Borgia F., Cannavò S.P., Altavilla D., Squadrito F. (2017). Differential Expression of Nitric Oxide Synthase Isoforms nNOS and iNOS in Patients with Non-Segmental Generalized Vitiligo. Int. J. Mol. Sci..

[38] Xiong Z., Jiang B., Wu P.-F., Tian J., Shi L.-L., Gu J., Hu Z.-L., Fu H., Wang F., Chen J.-G. (2011). Antidepressant Effects of a Plant-Derived Flavonoid Baicalein Involving Extracellular Signal-Regulated Kinases Cascade. Biol. Pharm. Bull..

[39] Shen P., Lin W., Deng X., Ba X., Han L., Chen Z., Qin K., Huang Y., Tu S. (2021). Potential Implications of Quercetin in Autoimmune Diseases. Front. Immunol..

[40] Guan C., Xu W., Hong W., Zhou M., Lin F., Fu L., Liu D., Xu A. (2015). Quercetin attenuates the effects of H_2_O_2_ on endoplasmic reticulum morphology and tyrosinase export from the endoplasmic reticulum in melanocytes. Mol. Med. Rep..

[41] Takekoshi S., Nagata H., Kitatani K. (2014). Flavonoids enhance melanogenesis in human melanoma cells. Tokai J. Exp. Clin. Med..

[42] Nagata H., Takekoshi S., Takeyama R., Homma T., Yoshiyuki Osamura R. (2004). Quercetin enhances melanogenesis by increasing the activity and synthesis of tyrosinase in human melanoma cells and in normal human melanocytes. Pigment Cell Res..

[43] Liu-Smith F., Meyskens F.L. (2016). Molecular mechanisms of flavonoids in melanin synthesis and the potential for the prevention and treatment of melanoma. Mol. Nutr. Food Res..

[44] Samad N., Saleem A., Yasmin F., Shehzad M.A. (2018). Quercetin Protects Against Stress-Induced Anxiety- and Depression-Like Behavior and Improves Memory in Male Mice. Physiol. Res..

[45] Guan Y., Wang J., Wu X., Song L., Wang Y., Gong M., Li B. (2021). Quercetin reverses chronic unpredictable mild stress-induced depression-like behavior in vivo by involving nuclear factor-E2-related factor 2. Brain Res..

[46] Silva Dos Santos J., Gonçalves Cirino J.P., de Oliveira Carvalho P., Ortega M.M. (2021). The Pharmacological Action of Kaempferol in Central Nervous System Diseases: A Review. Front. Pharmacol..

[47] Oh S.M., Kim Y.P., Chung K.H. (2006). Biphasic effects of kaempferol on the estrogenicity in human breast cancer cells. Arch. Pharmacal Res..

[48] Chuwa A.H., Sone K., Oda K., Tanikawa M., Kukita A., Kojima M., Oki S., Fukuda T., Takeuchi M., Miyasaka A. (2018). Kaempferol, a natural dietary flavonoid, suppresses 17β-estradiol-induced survivin expression and causes apoptotic cell death in endometrial cancer. Oncol. Lett..

[49] Tang H., Yang L., Wu L., Wang H., Chen K., Wu H., Li Y. (2021). Kaempferol, the melanogenic component of Sanguisorba officinalis, enhances dendricity and melanosome maturation/transport in melanocytes. J. Pharmacol. Sci..

[50] Gao W., Wang W., Peng Y., Deng Z. (2019). Antidepressive effects of kaempferol mediated by reduction of oxidative stress, proinflammatory cytokines and up-regulation of AKT/β-catenin cascade. Metab. Brain Dis..

[51] Ahmad H., Rauf K., Zada W., McCarthy M., Abbas G., Anwar F., Shah A.J. (2020). Kaempferol Facilitated Extinction Learning in Contextual Fear Conditioned Rats via Inhibition of Fatty-Acid Amide Hydrolase. Molecules.

[52] Zink A., Traidl-Hoffmann C. (2015). Green tea in dermatology–myths and facts. J. Dtsch. Dermatol. Ges..

[53] Chu C., Deng J., Man Y., Qu Y. (2017). Green Tea Extracts Epigallocatechin-3-gallate for Different Treatments. BioMed Res. Int..

[54] Di Bartolomeo L., Motolese A., Del Giudice M.M., Cuppari C., Ceravolo G., Chimenz R., Chimenz S., Sestito M., Vaccaro F., Borgia F. (2022). Papillomavirus skin infections and children: An overview on cutaneous and anogenital warts treatment. J. Biol. Regul. Homeost. Agents.

[55] Hu W., Zhang L., Lin F., Lei J., Zhou M., Xu A. (2021). Topical epigallocatechin-3-gallate in the treatment of vitiligo. Australas. J. Dermatol..

[56] Zhu Y., Wang S., Lin F., Li Q., Xu A. (2014). The therapeutic effects of EGCG on vitiligo. Fitoterapia.

[57] Ning W., Wang S., Liu D., Fu L., Jin R., Xu A. (2016). Potent effects of peracetylated (-)-epigallocatechin-3-gallate against hydrogen peroxide-induced damage in human epidermal melanocytes via attenuation of oxidative stress and apoptosis. Clin. Exp. Dermatol..

[58] Ning W., Wang S., Dong X., Liu D., Fu L., Jin R., Xu A. (2015). Epigallocatechin-3-gallate (EGCG) Suppresses the Trafficking of Lymphocytes to Epidermal Melanocytes via Inhibition of JAK2: Its Implication for Vitiligo Treatment. Biol. Pharm. Bull..

[59] Li G., Yang J., Wang X., Zhou C., Zheng X., Lin W. (2020). Effects of EGCG on depression-related behavior and serotonin concentration in a rat model of chronic unpredictable mild stress. Food Funct..

[60] Wang J., Li P., Qin T., Sun D., Zhao X., Zhang B. (2020). Protective effect of epigallocatechin-3-gallate against neuroinflammation and anxiety-like behavior in a rat model of myocardial infarction. Brain Behav..

[61] Vignes M., Maurice T., Lanté F., Nedjar M., Thethi K., Guiramand J., Récasens M. (2006). Anxiolytic properties of green tea polyphenol (−)-epigallocatechin gallate (EGCG). Brain Res..

[62] Loftis J.M., Wilhelm C., Huckans M. (2012). Effect of epigallocatechin gallate supplementation in schizophrenia and bipolar disorder: An 8-week, randomized, double-blind, placebo-controlled study. Ther. Adv. Psychopharmacol..

[63] Vaughn A.R., Branum A., Sivamani R.K. (2016). Effects of Turmeric (Curcuma longa) on Skin Health: A Systematic Review of the Clinical Evidence. Phytother. Res..

[64] Asawanonda P., Klahan S.-O. (2010). Tetrahydrocurcuminoid Cream Plus Targeted Narrowband UVB Phototherapy for Vitiligo: A Preliminary Randomized Controlled Study. Photomed. Laser Surg..

[65] Ashrafizadeh M., Ahmadi Z., Mohamamdinejad R., Farkhondeh T., Samarghandian S. (2020). Curcumin Activates the Nrf2 Pathway and Induces Cellular Protection Against Oxidative Injury. Curr. Mol. Med..

[66] Skyvalidas D., Mavropoulos A., Tsiogkas S., Dardiotis E., Liaskos C., Mamuris Z., Roussaki-Schulze A., Sakkas L.I., Zafiriou E., Bogdanos D.P. (2020). Curcumin mediates attenuation of pro-inflammatory interferon γ and interleukin 17 cytokine responses in psoriatic disease, strengthening its role as a dietary immunosuppressant. Nutr. Res..

[67] Tu C.-X., Lin M., Lu S.-S., Qi X.-Y., Zhang R.-X., Zhang Y.-Y. (2011). Curcumin Inhibits Melanogenesis in Human Melanocytes. Phytother. Res..

[68] Lv J., Yang Y., Jia B., Li S., Zhang X., Gao R. (2021). The Inhibitory Effect of Curcumin Derivative J147 on Melanogenesis and Melanosome Transport by Facilitating ERK-Mediated MITF Degradation. Front. Pharmacol..

[69] Sadeghian M., Rahmani S., Jamialahmadi T., Johnston T.P., Sahebkar A. (2020). The effect of oral curcumin supplementation on health-related quality of life: A systematic review and meta-analysis of randomized controlled trials. J. Affect. Disord..

[70] García-Gutiérrez M.S., Navarrete F., Gasparyan A., Austrich-Olivares A., Sala F., Manzanares J. (2020). Cannabidiol: A Potential New Alternative for the Treatment of Anxiety, Depression, and Psychotic Disorders. Biomolecules.

[71] Golub V., Reddy D.S. (2020). Cannabidiol Therapy for Refractory Epilepsy and Seizure Disorders. Cannabinoids Neuropsychiatr. Disord..

[72] Baswan S.M., Klosner A.E., Glynn K., Rajgopal A., Malik K., Yim S., Stern N. (2020). Therapeutic Potential of Cannabidiol (CBD) for Skin Health and Disorders. Clin. Cosmet. Investig. Dermatol..

[73] Atalay S., Jarocka-Karpowicz I., Skrzydlewska E. (2019). Antioxidative and Anti-Inflammatory Properties of Cannabidiol. Antioxidants.

[74] Magina S., Esteves-Pinto C., Moura E., Serrão M.P., Moura D., Petrosino S., Di Marzo V., Vieira-Coelho M.A. (2011). Inhibition of basal and ultraviolet B-induced melanogenesis by cannabinoid CB1 receptors: A keratinocyte-dependent effect. Arch. Dermatol. Res..

[75] Hwang Y.S., Kim Y.-J., Kim M.O., Kang M., Oh S.W., Nho Y.H., Park S.-H., Lee J. (2017). Cannabidiol upregulates melanogenesis through CB1 dependent pathway by activating p38 MAPK and p42/44 MAPK. Chem. Interactions.

[76] White C.M. (2019). A Review of Human Studies Assessing Cannabidiol’s (CBD) Therapeutic Actions and Potential. J. Clin. Pharmacol..

[77] Jung G.-D., Yang J.-Y., Song E.-S., Park J.-W. (2001). Stimulation of melanogenesis by glycyrrhizin in B16 melanoma cells. Exp. Mol. Med..

[78] Chrzanowski J., Chrzanowska A., Graboń W. (2020). Glycyrrhizin: An old weapon against a novel coronavirus. Phytother. Res..

[79] Liu X., Zhuang J., Wang D., Lv L., Zhu F., Yao A., Xu T. (2019). Glycyrrhizin suppresses inflammation and cell apoptosis by inhibition of HMGB1 via p38/p-JUK signaling pathway in attenuating intervertebral disc degeneration. Am. J. Transl. Res..

[80] Vaccaro M., Cannavò S.P., Imbesi S., Cristani M., Barbuzza O., Tigano V., Gangemi S. (2014). Increased serum levels of interleukin-23 circulating in patients with non-segmental generalized vitiligo. Int. J. Dermatol..

[81] Mou K., Pan W., Han D., Wen X., Cao F., Miao Y., Li P. (2019). Glycyrrhizin protects human melanocytes from H_2_O_2_-induced oxidative damage via the Nrf2-dependent induction of HO-1. Int. J. Mol. Med..

[82] Lee J., Jung E., Park J., Jung K., Park E., Kim J., Hong S., Park J., Park S., Lee S. (2005). Glycyrrhizin Induces Melanogenesis by Elevating a cAMP Level in B16 Melanoma Cells. J. Investig. Dermatol..

[83] Mou K., Han D., Liu W., Li P. (2016). Combination therapy of orally administered glycyrrhizin and UVB improved active-stage generalized vitiligo. Braz. J. Med. Biol. Res..

[84] Cao Z.-Y., Liu Y.-Z., Li J.-M., Ruan Y.-M., Yan W.-J., Zhong S.-Y., Zhang T., Liu L.-L., Wu R., Wang B. (2020). Glycyrrhizic acid as an adjunctive treatment for depression through anti-inflammation: A randomized placebo-controlled clinical trial. J. Affect. Disord..

[85] Wu T.-Y., Liu L., Zhang W., Zhang Y., Liu Y.-Z., Shen X.-L., Gong H., Yang Y.-Y., Bi X.-Y., Jiang C.-L. (2015). High-mobility group box-1 was released actively and involved in LPS induced depressive-like behavior. J. Psychiatr. Res..

[86] Hisaoka-Nakashima K., Tomimura Y., Yoshii T., Ohata K., Takada N., Zhang F.F., Nakamura Y., Liu K., Wake H., Nishibori M. (2019). High-mobility group box 1-mediated microglial activation induces anxiodepressive-like behaviors in mice with neuropathic pain. Prog. Neuro-Psychopharmacol. Biol. Psychiatry.

[87] Girard J.-P. (2007). A Direct Inhibitor of HMGB1 Cytokine. Chem. Biol..

[88] Vaccaro M., Cicero F., Mannucci C., Calapai G., Spatari G., Barbuzza O., Cannavò S.P., Gangemi S. (2016). IL-33 circulating serum levels are increased in patients with non-segmental generalized vitiligo. Arch. Dermatol. Res..

[89] Lai S., Shi L., Jiang Z., Lin Z. (2019). Glycyrrhizin treatment ameliorates post-traumatic stress disorder-like behaviours and restores circadian oscillation of intracranial serotonin. Clin. Exp. Pharmacol. Physiol..

